# Metabolic Investigations of the Molecular Mechanisms Associated with Parkinson’s Disease

**DOI:** 10.3390/metabo7020022

**Published:** 2017-05-24

**Authors:** Robert Powers, Shulei Lei, Annadurai Anandhan, Darrell D. Marshall, Bradley Worley, Ronald L. Cerny, Eric D. Dodds, Yuting Huang, Mihalis I. Panayiotidis, Aglaia Pappa, Rodrigo Franco

**Affiliations:** 1 Department of Chemistry, University of Nebraska-Lincoln, Lincoln, NE 68588, USA; shulei@huskers.unl.edu (S.L.); darrell.marshall@huskers.unl.edu (D.D.M.); bradley.worley@huskers.unl.edu (B.W.); rcerny1@unl.edu (R.L.C.); eric.dodds@unl.edu (E.D.D.); yuting.huang@huskers.unl.edu (Y.H.); 2 Department of Biochemistry, University of Nebraska-Lincoln, Lincoln, NE 68588, USA; 3 Redox Biology Center, University of Nebraska-Lincoln, Lincoln, NE 68588, USA; ananbiochem@gmail.com; 4 School of Veterinary Medicine and Biomedical Sciences, University of Nebraska-Lincoln, Lincoln, NE 68588, USA; 5 Department of Applied Sciences, Northumbria University, Newcastle Upon Tyne NE1 8ST, UK; m.panagiotidis@northumbria.ac.uk; 6 Department of Molecular Biology and Genetics, Democritus University of Thrace, Alexandroupolis 68100, Greece; apappa@mbg.duth.gr

**Keywords:** Parkinson’s Disease, genetics, toxin synergy, molecular mechanisms, NMR, mass spectrometry

## Abstract

Parkinson’s disease (PD) is a neurodegenerative disorder characterized by fibrillar cytoplasmic aggregates of α-synuclein (i.e., Lewy bodies) and the associated loss of dopaminergic cells in the substantia nigra. Mutations in genes such as α-synuclein (*SNCA*) account for only 10% of PD occurrences. Exposure to environmental toxicants including pesticides and metals (e.g., paraquat (PQ) and manganese (Mn)) is also recognized as an important PD risk factor. Thus, aging, genetic alterations, and environmental factors all contribute to the etiology of PD. In fact, both genetic and environmental factors are thought to interact in the promotion of idiopathic PD, but the mechanisms involved are still unclear. In this study, we summarize our findings to date regarding the toxic synergistic effect between α-synuclein and paraquat treatment. We identified an essential role for central carbon (glucose) metabolism in dopaminergic cell death induced by paraquat treatment that is enhanced by the overexpression of α-synuclein. PQ “hijacks” the pentose phosphate pathway (PPP) to increase NADPH reducing equivalents and stimulate paraquat redox cycling, oxidative stress, and cell death. PQ also stimulated an increase in glucose uptake, the translocation of glucose transporters to the plasma membrane, and AMP-activated protein kinase (AMPK) activation. The overexpression of α-synuclein further stimulated an increase in glucose uptake and AMPK activity, but impaired glucose metabolism, likely directing additional carbon to the PPP to supply paraquat redox cycling.

## 1. Introduction

### 1.1. Parkinson’s Disease Overview

**Figure 1:**
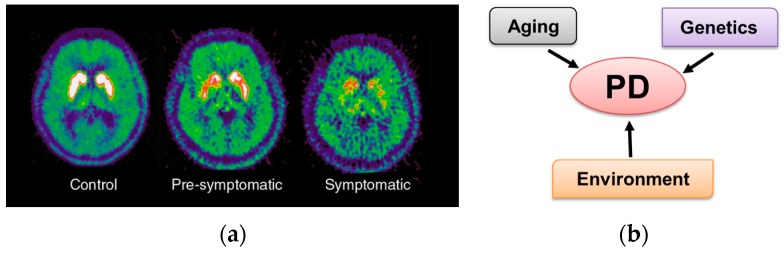
Parkinson’s disease results from dopaminergic neuron cell death in the substantia nigra: (**a**) In vivo imaging of dopaminergic activity in the Parkinsonian basal ganglia shown by [^18^F] fluorodopa PET. The signal from striatum in a healthy control subject, a patient with symptomatic Parkinson’s disease and a twin who was asymptomatic at the time of scan but who subsequently developed the disease. Reproduced with permission from [3]. (**b**) Schematic of the multiple factors that contribute to the development of Parkinson’s disease. Both aging and environmental factors modify epigenetic patterns, which may also enhance an individual’s likelihood of developing PD.

Parkinson’s disease (PD) affects over one million individuals in the United States and more than 10 million people worldwide [1]. PD is a chronic progressive neurodegenerative disorder that leads to shaking (tremors) and difficulty with walking, movement, and coordination. Currently, there is no cure for PD or drug to stop the progression of the disease, but there are treatments to manage symptoms [2]. PD is associated with the loss of dopaminergic neurons from the substantia nigra pars compacta within the midbrain (Figure 1a) [3,4]. The death of these dopaminergic neurons leads to a deficiency of dopamine in the caudate and putamen (“striatum”), which results in an observed loss of muscle control. In addition to neuron loss, PD is also characterized by the presence of Lewy bodies—protein aggregates within neurons [5]. The exact cause of PD is currently unknown, but age is an important risk factor [6]. Individuals over the age of 60 are twice as likely to develop PD relative to the general population. Only about 10% of PD cases have a family history of the disease, and, to date, 18 genetic mutations (*PARK1*, *PARK2*, etc.) have been putatively linked to PD [7,8]. Genetic alterations in α-synuclein [9,10], Parkin [11,12], DJ-1 [13], PINK1 [11], and LRRK2 [14] have been associated with approximately 3 to 5% of PD. Environmental factors have also been linked to an increase in the incidence of PD or risk for developing PD [15]. In fact, sporadic or idiopathic PD is linked to genetic alterations and occupational or environmental factors. Exposure to pesticides, heavy metals, infectious agents, industrialization, and/or dietary factors has been associated with an increased occurrence of PD. Recent studies have demonstrated that environmental exposures modify DNA methylation patterns, chromatin structure, and non-coding RNA signaling (epigenetics), which might contribute to the individual’s susceptibility to developing PD. Epigenetic patterns defined during aging and development can be altered by environmental exposures [16,17,18]. Paraquat induces epigenetic changes by promoting histone acetylation [19] and, conversely, paraquat toxicity has been reported to be enhanced by inhibition of DNA methyltransferases [20]. Thus, PD appears to be multifactorial where a combination of age, genetics, and environmental factors contributes to disease development (Figure 1b) [21]. 

### 1.2. PD and Environmental Risk Factors

The largest epidemiology study of PD in the USA identified a number of environmental factors correlated with an increased incidence of PD [22]. Specifically, PD was found to be more common in the Midwest and the Northeast. In fact, the state of Nebraska was observed to have the highest prevalence of PD in the world (Table 1). Again, this is consistent with areas associated with agriculture and metal processing having high rates of PD. Thus, prolonged exposure to herbicides or insecticides from farming or metals from industry likely contributes to PD. Consequently, paraquat (herbicide), rotenone (insecticide), 6-hydroxydopamine (6-OHDA, neurotoxin), 1-methyl-4-phenylpyridinium (MPP^+^, herbicide), 1-methyl-4-phenyl-1,2,3,6-tetrahydropyridine (MPTP, prodrug), and manganese have all been shown to induce PD-like symptoms. These compounds are routinely and interchangeably used to induce dopaminergic neuron cell death and as chemical mimics of PD in animal models. MPTP was discovered as a contaminant in illicit opioid synthesis, and acts as a prodrug that is converted to MPP^+^ in the brain and is selectively taken up by dopaminergic cells [23,24]. MPP^+^ inhibits mitochondrial respiratory complex I of the electron transport chain and interferes with oxidative phosphorylation in the mitochondria [25,26]. Paraquat has a structure similar to MPTP, but is a poor inhibitor of complex I. Instead, paraquat toxicity has been primarily attributed to its redox cycling that generates reactive oxygen species (ROS). Paraquat is reported to induce dopaminergic degeneration in vivo, which is one of the pathological hallmarks of PD, but contradictory results exist as well. While some environmental toxicants linked to PD such as PQ have been demonstrated to have a limited capacity to diffuse across the blood–brain barrier (BBB) [27,28], a significant increase in the permeability of the BBB in the postcommissural putamen of PD patients has been reported [29] and breakdown of the BBB has been shown to occur during aging [30]. Interestingly, α-synuclein impairs tight junction protein expression [30]. These findings again suggest the likelihood that neurodegeneration linked to environmental exposure is a consequence of genetics and or aging converging to promote dopaminergic cell loss.

The chemical similarity between paraquat and MPTP initiated an investigation into an agricultural link with PD. Consequently, a correlation between paraquat agricultural usage and PD rates has been observed. The naturally occurring insecticide rotenone also inhibits complex I, which leads to energy failure and cell death [31,32]. Similarly, 6-OHDA has been proposed to induce dopaminergic neuron cell death by producing pro-oxidant capacity and selective uptake via dopamine transporters [23,33,34].

Regardless of the environmental toxin, there appears to be a common mechanism that leads to dopaminergic neuron cell death. Neurons have a very high energy demand and high glucose usage. Consequently, alterations in energy metabolism have been reported in early PD. Specifically, environmental toxins alter redox homeostasis, energy metabolism, and central carbon metabolism. Environmental toxins appear to increase ROS either through a direct redox cycling or indirectly by inhibiting the electron transport chain. Consequently, this leads to dysfunctional mitochondria and cell death. Thus, toxin-induced alterations in metabolic pathways important to central carbon metabolism, energy metabolism and redox homeostasis present a clear role for metabolomics in investigating PD.

### 1.3. Lewy Bodies and α-Synuclein

**Figure 2:**
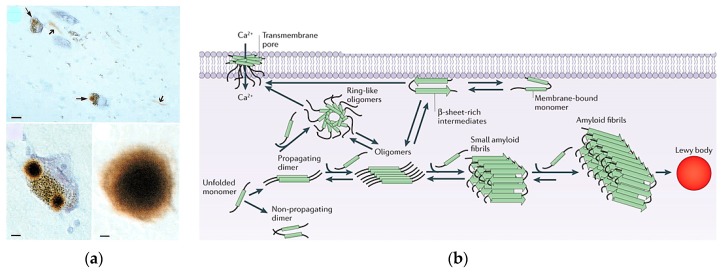
(**a**) Substantia nigra from patients with Parkinson’s disease (from the MRC Cambridge Brain Bank) immunostained for α-synuclein. (**Top**) Two pigmented nerve cells, each containing an α-synuclein-positive Lewy body (long arrows). Lewy neurites (short arrows) are also immunopositive. Scale bar, 20 μm. (**Bottom Left**) A pigmented nerve cell with two α-synuclein-positive Lewy bodies. Scale bar, 8 μm. (**Bottom Right**) α-Synuclein-positive, extracellular Lewy body. Scale bar, 4 μm. Reproduced with permission from [38]. (**b**) α-synuclein (α-syn) aggregation can take place either in the cytoplasm or in association with the cellular membrane. In the cytosol, unfolded monomers interact to form initially unstable dimers, which grow slowly to generate oligomers of varying morphologies—including transient spherical and ring-like oligomers—that eventually convert to fibrils. The α-syn oligomers are in equilibrium with monomers and convert to fibrils by monomer addition via a nucleated polymerization mechanism. The accumulation of these amyloid fibrils leads to the formation of intracellular inclusions called Lewy bodies. Membrane-bound monomeric α-syn adopts a predominantly α-helical confirmation, but at high concentrations the protein undergoes a conformational change either before or coincident with its oligomerization to form membrane-bound β-sheet-rich structures that self-associate to form oligomers, including trans-membrane amyloid pores (the formation of which may involve several intermediates) and fibrils. Note that the ring-like cytosolic oligomers may also associate with the membrane and form trans-membrane pores. During α-syn fibrillogenesis and aggregation, the intermediate species (oligomers and amyloid fibrils) are highly toxic, affecting mitochondrial function, endoplasmic reticulum–Golgi trafficking, protein degradation and/or synaptic transmission, and these intracellular effects are thought to induce neurodegeneration. The transmembrane pores disrupt membrane integrity as well as intracellular calcium homeostasis and signaling, and may also contribute to neuronal toxicity. Interestingly, α-syn oligomers and fibrils, as well as the monomers, can be transferred between cells and induce disease spreading to other brain regions. Spreading mechanisms are multiple and can occur via endocytosis, direct penetration, trans-synaptic transmission, or membrane receptors. Once inside the host cells, α-syn aggregates can nucleate aggregation and propagate via the mechanisms described above. Reproduced with permission from [39].

A hallmark of PD is the formation of intracellular protein aggregates or Lewy bodies in the dopaminergic neurons within the substantia nigra (Figure 2a) [36]. Lewy bodies are found in the cytoplasm as single or multiple spherical masses consisting of a dense protein core surrounded by a pale halo. Lewy bodies have a filamentous structure and contain over 70 different biological molecules falling within 10 distinct classes. α-Synuclein is a major component of Lewy bodies and forms the fibrils (Figure 2b) [37,38]. In addition to α-synuclein fibrils, other components of Lewy bodies correspond to proteins involved in: (i) α-synuclein binding; (ii) synphilin-1-binding; (iii) ubiquitin-proteasome system; (iv) cellular responses; (v) phosphorylation and signal transduction; (vi) cytoskeleton; and (vii) the cell cycle. Importantly, Lewy bodies are correlated with neuronal loss and cognitive impairment, which suggests that neurons that contain Lewy bodies are dying. However, there is no evidence that Lewy bodies are the actual cause of cell death. In fact, Lewy bodies may be a cytoprotective mechanism in PD, while α-synuclein protofibrils might be cytotoxic agents. Lewy bodies may function to sequester and degrade the α-synuclein fibrils. 

**Figure 3:**
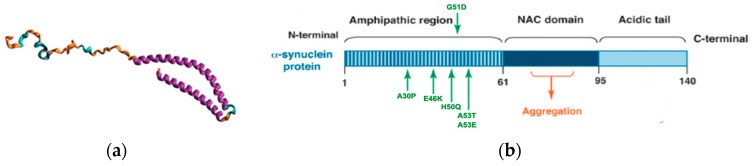
Structure of α-synuclein: (**a**) Schematic representation of micelle-bound α-synuclein (α-syn; Protein Data Bank ID: 1XQ8) [40]. The N-terminal region, the non-amyloid-β component of Alzheimer’s disease amyloid plaques (NAC) region and the C-terminal part are colored blue, orange and red, respectively. Numbers refer to amino acid residues flanking the different regions. Reproduced with permission from [39]. (**b**) α-Synuclein protein domain structure. α-Syn is a 140-amino-acid protein and its sequence can be divided into three regions with distinct structural characteristics. The highly conserved N-terminal domain encodes for a series of imperfect 11 amino acid repeats with a consensus motif of KTKEGV reminiscent of the lipid-binding domain of apolipoproteins, which in certain conditions forms amphipathic helices. The six missense mutations known to cause familial PD (A30P, E46K, H50Q, G51D, A53E, and A53T) lie in the amphipathic region, suggesting an important function for this region of the protein. The central hydrophobic region (non-amyloid-β component or NAC domain) of α-synuclein is associated with an increased propensity of the protein to form fibrils [62]. The acidic C-terminal tail contains mostly negatively charged residues and is largely unfolded. Reproduced with permission from [63].

α-Synuclein is a 140-amino-acid (14.5 kDa) protein of unknown function that is a natively unstructured monomer (Figure 3a) [39,40]. However, there has been some progress in revealing a role for α-synuclein in vesicle trafficking, synaptic vesicles endocytosis, and chaperoning of the SNARE complex assembly [41,42,43]. The protein consists of three distinct domains: (i) an amphipathic lysine-rich N-terminus (1–60) that interacts with membranes and forms an α-helix; (ii) an acidic disordered C-terminus (96–140) that is postulated to regulate nuclear localization and is involved in interactions with metals, small molecules and other proteins; and (iii) a hydrophobic central region (61–95), which is critical for protein aggregation and is commonly referred to as the non-amyloid-β component of AD amyloid plaques (NAC) (Figure 3b). α-Synuclein is an abundant neuronal protein (~1% of cytosol proteins) that is expressed throughout the brain with particularly high levels in the substantia nigra. α-Synuclein is primarily located in the presynaptic terminal of neurons. Thus, the protein may play a role in regulating the release of dopamine or the supply of synaptic vesicles. Genetic mutations in α-synuclein (*SNCA*) [9,10] or overexpression due to multiplication of *SNCA* [44] have been associated with familial and sporadic PD (Figure 3b). Oligomerization of α-synuclein and the resulting fibril formation is central to the pathogenesis of PD (Figure 2b). α-Synuclein aggregates have been shown to bind lipid membranes, to form pore-like structures, and to increase membrane permeability [45,46,47,48]. The resulting α-synuclein aggregates cause neuroinflammation, neurodegeneration, and neuronal cell death. A variety of factors including oxidative stress [49], post-translational modifications [50,51,52], proteolysis [53,54]; and fatty acids [55,56,57], phospholipids [55,58], and metal ion [59,60] concentrations have been shown to affect α-synuclein aggregation. Mitochondrial dysfunction and energy failure induced by environmental toxicants may also lead to α-synuclein misfolding and aggregation through impairment in the protein quality control mechanisms. Importantly, there is a growing body of evidence that indicates that α-synuclein is localized to the mitochondria under both normal and stress conditions [61]. α-Synuclein may play a role in regulating mitochondrial function, in which either the overexpression or lose of α-synuclein function may result in mitochondrial damage and cell death. So, α-synuclein also plays a prominent role in idiopathic PD. Again, PD incidences may increase from combined genetic and environmental factors. 

## 2. NMR and MS Metabolomics Protocol to Investigate PD

### 2.1. Combining NMR and MS Improves Coverage of the Metabolome

Nuclear magnetic resonance (NMR) [64] and mass spectrometry (MS) [65] have been the primary analytical tools used to obtain metabolomics datasets. Historically, only NMR or MS has been used for a given metabolomics study, in which the choice of instrumentation has been primarily decided upon based on an investigator’s experience and expertise, instead of the needs of the study. Consequently, a suboptimal analysis of the metabolome is likely to occur. In fact, NMR and MS are inherently complementary and when employed together provide a broader and more accurate coverage of the metabolome [66,67]. While the number of studies is still limited, a few projects that have used both NMR and MS have observed a common trend [68,69,70]. A set of metabolites was only observable by NMR, while a second set of metabolites was only detected by MS. A smaller subset of metabolites was observed by both NMR and MS. Simply, NMR only observes the most abundant metabolites (≥1 µM) and MS only observes the metabolites that readily ionize. There are other important differences between NMR and MS. NMR requires minimal sample handling before data collection, is easily quantifiable, and provides multiple means of metabolite identification. In addition to the higher sensitivity, (femtomolar to attomolar), MS also has a higher resolution (~10^3^–10^4^) and dynamic range (~10^3^–10^4^). However, chromatography is commonly required for MS because of the relatively narrow nominal mass and mass defect distribution of the metabolome [71]. The use of chromatography has its own limitations and may also lead to a loss of observable metabolites for a variety of reasons [72,73,74,75,76]. Simply, NMR and MS have unique sets of strengths and limitations and both analytical methods beneficially contribute to a metabolomics study. In fact, a number of recent methods highlight the benefits of combining NMR and MS to improve the accuracy of metabolite identification or for identifying unknown metabolites [77,78,79,80,81].

### 2.2. Combined NMR and MS Metabolomics Methodology

**Figure 4:**
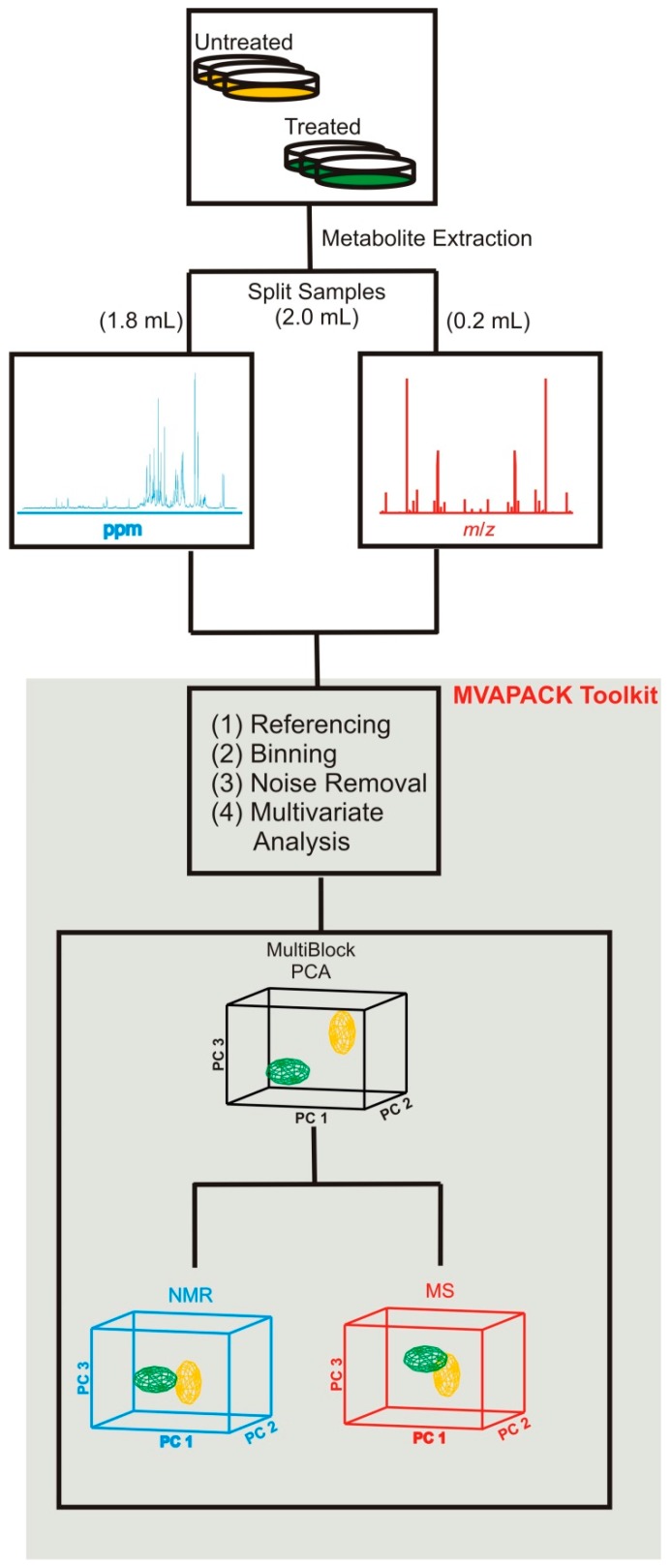
A flow chart illustrating our protocol for combining NMR and MS datasets for metabolomics. 2.0 mL of a single metabolite extract was split into 1.8 mL and 0.2 mL for NMR and MS analysis, respectively. Spectral binning of the NMR data used adaptive intelligent binning. For MS, the background is first subtracted before spectral binning. Spectral binning of the MS data used fixed binning with a set bin width of 0.5 *m/z*. Noise removal and normalization were separately applied to the NMR and MS datasets. The NMR and MS datasets were then modeled by MB-PCA and MB-PLS. The resulting block scores and loadings are then analyzed for significantly contributing metabolites. Reproduced with permission from [82].

Towards this end, we recently optimized sample preparation, data collection, and data handling protocols to effectively integrate direct-infusion electrospray ionization mass spectrometry (DI-ESI-MS) data with 1D ^1^H NMR spectra (Figure 4) [82,83]. By splitting metabolite extracts optimized for NMR acquisition and by diluting the MS-bound aliquots tenfold in H_2_O/methanol/formic acid (49.57 : 49.75 : 0.5), we obtained samples suitable for NMR and DI-ESI-MS while avoiding chromatographic separations. We also optimized several DI-ESI-MS ion source conditions to maximize the quality of the MS metabolomics data: sampling cone voltage (SCV) of 40 V, extraction voltage (ECV) of 4.0 V, desolvation temperature of 150 °C, desolvation gas flow of 500 L/h, and a cone gas flow of 0 L/h. We preprocessed the acquired mass spectra with background subtraction, followed by uniform binning with a 0.5 *m/z* bin size and spectral noise region removal. NMR spectra were processed with our MVAPACK [84] software and automatically phased and normalized using our phase-scatter correction (PSC) algorithm [85]. Chemical shift regions containing spectral baseline noise or solvent signals were removed based on our previously developed protocols [86,87]. Binning was performed using an adaptive intelligent binning algorithm [88] implemented in MVAPACK [84] that minimizes the splitting of signals between multiple bins. 

**Figure 5:**
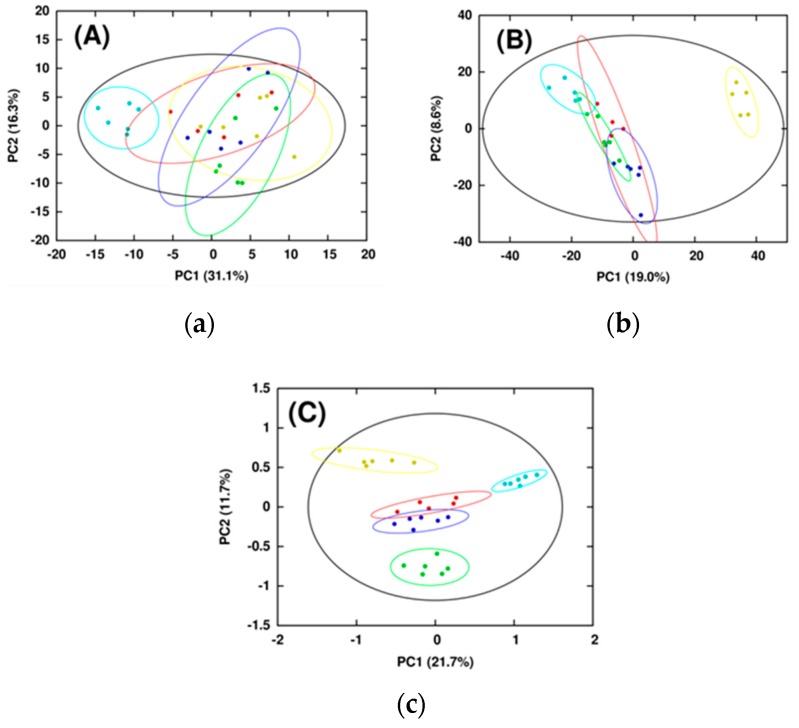
Scores generated from (**a**) PCA of 1D ^1^H NMR spectra; (**b**) PCA of DI-ESI-MS spectra; and (**c**) MB-PCA of 1D ^1^H NMR and DI-ESI-MS spectra of metabolomes extracted from human dopaminergic neuroblastoma cells treated with environmental/mitochondrial toxins. The ellipses in the PCA score plots correspond to the 95% confidence limits from a normal distribution for each cluster. Symbols designate the following classes: Control (●); Rotenone (●); 6-OHDA (●); MPP+ (●); and Paraquat (●). Reproduced with permission from [82].

Integrating MS and NMR data clearly resulted in better class separation and tighter within-class variation than using only NMR or MS datasets (Figure 5). This was accomplished by incorporating multivariate statistical techniques to properly handle multiple analytical datasets [89,90,91]—multiblock principal component analysis (MB-PCA) and multiblock partial least squares (MB-PLS)—into MVAPACK [84]. Multiblock methods are similar to traditional PLS and PCA, but provide a means for analyzing data from multiple analytical sources [89,90,91]. Simply put, the NMR and MS spectral data are placed into separate “blocks,” which allows for the generation and simultaneous usage of within-block and between-block data correlations. The inclusion of MB-PLS also led to the use of backscaled loadings to identify biologically important metabolites that contributed significantly to group separation. Importantly, these NMR and MS spectral changes are now identified as being statistically correlated. In addition to MB-PCA and MB-PLS, the data were jointly modeled using multiblock orthogonal projections to latent structures (MB-OPLS), which corroborated the MB-PLS analysis while better differentiating group separations [92]. By effectively integrating NMR and MS datasets, we could thoroughly analyze the metabolic changes to human dopaminergic cells resulting from treatments with toxins that were not achievable with just the NMR or MS data.

## 3. PD and Mitochondrial/Environmental Toxins

### 3.1. Paraquat Induces Unique Metabolic Changes

In a previous study, human dopaminergic neuroblastoma cells (SK-N-SH) were treated with sublethal doses of environmental toxins known to induce dopaminergic cell death. Specifically, cells were treated with MPP^+^, 50 µM 6-OHDA, 0.5 mM paraquat, or 4.0 µM rotenone for 24 h [82,83]. The metabolome was extracted as described above (Figure 4) and analyzed with both 1D ^1^H NMR and DI-ESI-MS. The resulting MB-PCA model (Figure 5c) was valid based on both a CV-ANOVA *p* value of 1.7 × 10^−12^ and response permutation testing that yielded a *p* value equal to zero. The MB-PCA model yielded a clear separation between the control group and the four toxin treatments. Furthermore, the control and paraquat groups were separated from the other toxin treatments. Also, the MPP^+^ treatment group was significantly separated from 6-OHDA and rotenone treatments. Again, these group separations were not apparent if only the NMR or MS dataset was used (Figure 5). More importantly, 6-OHDA, MPP^+^, paraquat, and rotenone have been routinely used as experimental models of PD since they all result to a certain and variable extent in dopaminergic neuron cell death and PD-like symptoms in animal models [93]. Nevertheless, our analysis clearly indicates that the metabolic impact of these four toxins is unique and, consequently, the molecular mechanism that results in neuronal cell death must be different. Since paraquat treatment resulted in the largest metabolome changes relative to untreated controls, we focused our further investigation on the molecular mechanism of PD on paraquat. 

**Figure 6:**
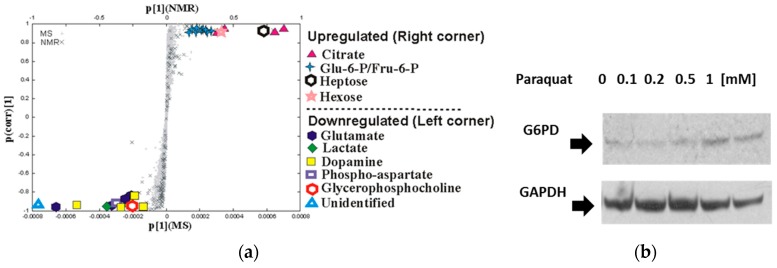
(**a**) Alterations in citrate, glucose 6-phosphate/fructose 6-phosphate, lactate, and glucose content are specific for paraquat treatment. S-plot was generated from the combined MB-PLS-DA of 1D ^1^H NMR spectra and DI-ESI-MS spectra. The S-plot was used to identify metabolites that significantly contribute to the class separation between untreated controls and paraquat treatment. The metabolites located in the upper right quadrant increased significantly while those located in the lower left quadrant significantly decreased after paraquat exposure. Reproduced with permission from [83]. (**b**) Western blot analysis of changes in glucose-6-phosphate dehydrogenase (G6PD) expression induced by paraquat. Paraquat induces an increase in the expression levels of G6PD. Glyceraldehyde 3-phosphate dehydrogenase (GAPDH) levels are used as loading controls for WBs. The changes observed in GAPDH levels might reflect of overall cell death (overall decrease in protein content) induced by PQ. Reproduced with permission from [83].

**Figure 7:**
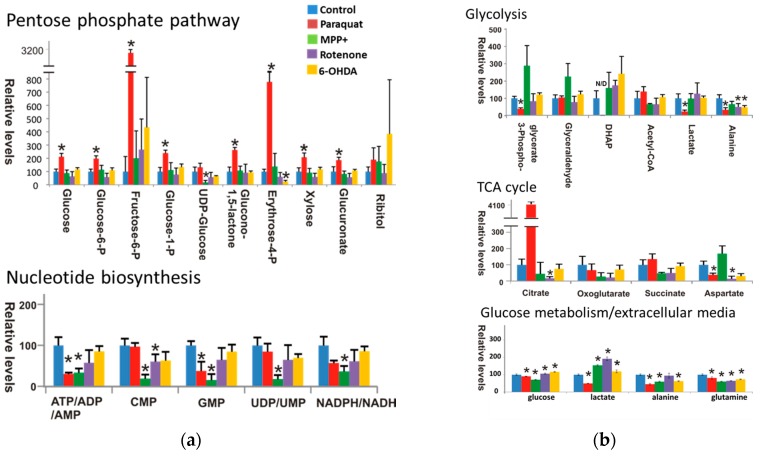
Paraquat induces selective changes in glucose metabolism, TCA cycle, and the PPP pathway. Cells were treated with paraquat (0.5 mM), rotenone (4 µM), MPP^+^ (2.5 mM), or 6-OHDA (50 µM) for 24 h in a glucose-free medium supplemented with ^13^C-glucose (3.5 g/L). Analysis of 2D ^1^H-^13^C HSQC NMR spectra was used to evaluate changes in glucose-derived metabolites. Bar graphs indicate the relative changes in peak intensity (concentration) for metabolites associated with the (**a**) PPP and nucleotide biosynthesis and (**b**) glycolysis, the TCA cycle, and metabolites found accumulated in the extracellular media. Data represent means ± SD of 3 independent experiments. * *p* < 0.05, control vs. neurotoxin treatments. ATP/ADP/AMP, ATP or ADP or AMP; DHAP, Dihydroxyacetone phosphate; NADP/NADPH, NADP or NADPH; UDP/UMP, UDP or UMP. Reproduced with permission from [83].

A detailed analysis of the metabolic changes by NMR and MS verified that paraquat uniquely perturbed the metabolome of dopaminergic neurons. For example, the S-plot (Figure 6a) generated from the MB-PLS-DA model identified metabolites significantly perturbed in dopaminergic neurons following paraquat treatment. Specifically, an increase in citrate, glucose 6-phosphate/fructose 6-phoshate, heptose (sedoheptulose), and hexose (glucose or myoinositol), and a decrease in lactate, glutamate, dopamine, and phospho-aspartate were clearly observed. A similar comparison was made between paraquat and the other toxins to determine if these metabolomic changes were unique to paraquat. The resulting Shared and Unique Structures (SUS) plot (data not shown), which is a union of two S-plots (paraquat vs. controls, and paraquat vs. MPP+, rotenone and 6-OHDA), verified that the changes in citrate, glucose 6-phosphate/fructose 6-phoshate, hexose, lactate, and dopamine were all a unique neuronal response to paraquat. Metabolome changes were further characterized by monitoring changes in the distribution and incorporation of ^13^C-carbons into metabolites (Figure 7). This was accomplished by interrogating intensity changes in 2D ^1^H-^13^C HSQC experiments after the addition of ^13^C glucose to the cell culture medium. Paraquat treatment resulted in an increase in glucose, glucose 6-phosphate, fructose-6-phosphate, glucose-1-phosphate, and glucono-1,5-lactone, which are associated with the pentose phosphate pathway (PPP). Importantly, these metabolic changes are consistent with the observed changes in the 1D ^1^H NMR and DI-ESI-MS spectral data. Paraquat also decreased purine levels (ATP, ADP, and AMP) and metabolites associated the glycolytic pathway [3-phospho glycerate, dihydroxyacetone phosphate (DHAP or glycerone phosphate), lactate, and alanine]. A decrease in extracellular glucose, lactate, and alanine was also observed. Furthermore, a large increase in citrate and a decrease in aspartate were observed following paraquat treatment. This is consistent with the inhibition of aconitase by paraquat-induced superoxide anion formation [94], since aconitase converts citrate to isocitrate in the tricarboxylic acid cycle (TCA). Also, aspartate is generated from oxaloacetate, which is produced from the TCA cycle. Finally, paraquat treatment also resulted in a decrease in total reduced (GSH) and oxidized (GSSG) glutathione. Critically, GSH depletion is an important contributor to oxidative stress and dopaminergic cell death [95]. Also, a decrease in GSH is one of the earliest biochemical alterations detected in incidental Lewy body disease, which is considered an asymptomatic precursor to PD [96]. A flow cytometry experiment confirmed a 76.4% decrease in GSH 48 h after treatment with paraquat. Also, cell death was correlated with the GSH decrease. In total, the observed metabolite changes by NMR and MS demonstrate that paraquat increased PPP metabolite accumulation while decreasing glycolysis and impairing the TCA cycle.

### 3.2. Glucose 6-Phosphate Dehydrogenase Regulates Paraquat Toxicity

Metabolomics is a valuable tool of systems biology and provides a unique and complementary view relative to traditional cellular assays and molecular biology data. In this context, the value and validity of metabolomics data are greatly enhanced when combined with other experimental results. Thus, our metabolomics data were supplemented with a proteomics analysis following 24-h treatment of human dopaminergic neuroblastoma cells with paraquat. As commonly observed with proteomics data, a number of proteins across a variety of biological processes exhibited significant upregulation or downregulation due to exposure to paraquat. Consequently, it is difficult to ascertain which changes in protein expression levels are primary responses to paraquat toxicity and which are downstream or secondary effects. However, by combining the metabolomics and proteomics data it was possible to identify overlapping metabolic processes that likely represent major biological responses to paraquat toxicity. The proteomics analysis identified increases in glucose 6-phosphate dehydrogenase (G6PD), mitochondrial malate dehydrogenase, phosphoglycerate kinase 1 (PGK1), ATP-citrate synthase (CS), and pyruvate kinases isozymes M1/M2 and a decrease in lactate dehydrogenase A/B chains, which all correlate with the alterations in the PPP, TCA cycle, and glycolysis pathway identified from the metabolomics data. G6PD was of particular interest since it is a rate-limiting enzyme of the PPP and a major source of NADPH [97]. The proteomics data were confirmed by Western blot (Figure 6b) and indicate that G6PD expression increased proportional to an increase in paraquat dosage. Thus, the metabolomics and proteomics data indicated that alterations in PPP and G6PD activity may be a result of paraquat toxicity.

### 3.3. Paraquat Hijacks the Pentose Phosphate Pathway

**Figure 8:**
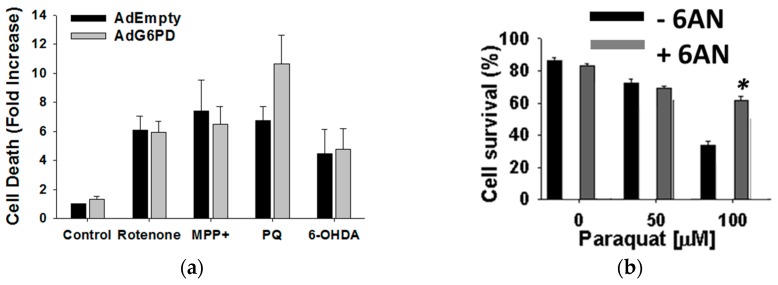
Paraquat-induced cell death is selectively regulated by glucose 6-phosphate dehydrogenase and the pentose phosphate pathway. (**a**) Cell death induced by paraquat (0.5 mM), rotenone (4 µM), MPP+ (2.5 mM) or 6-OHDA (50 µM) after 48 h of treatment, was simultaneously evaluated by flow cytometry using PI and mBCl. Cell death is observed as an increase in PI uptake. Data is represented as fold increase in the mean PI fluorescence and are means ± SE of three independent experiments. * *p* < 0.05, Empty vs. G6PD values. Reproduced with permission from [83]. (**b**) Cell death induced by paraquat was evaluated in the presence or absence of 6-aminonicotinamide (6-AN, 1 mM). Cell death is represented as an increase in the population of cells (%) with increased PI fluorescence. Reproduced with permission from [98].

**Figure 9:**
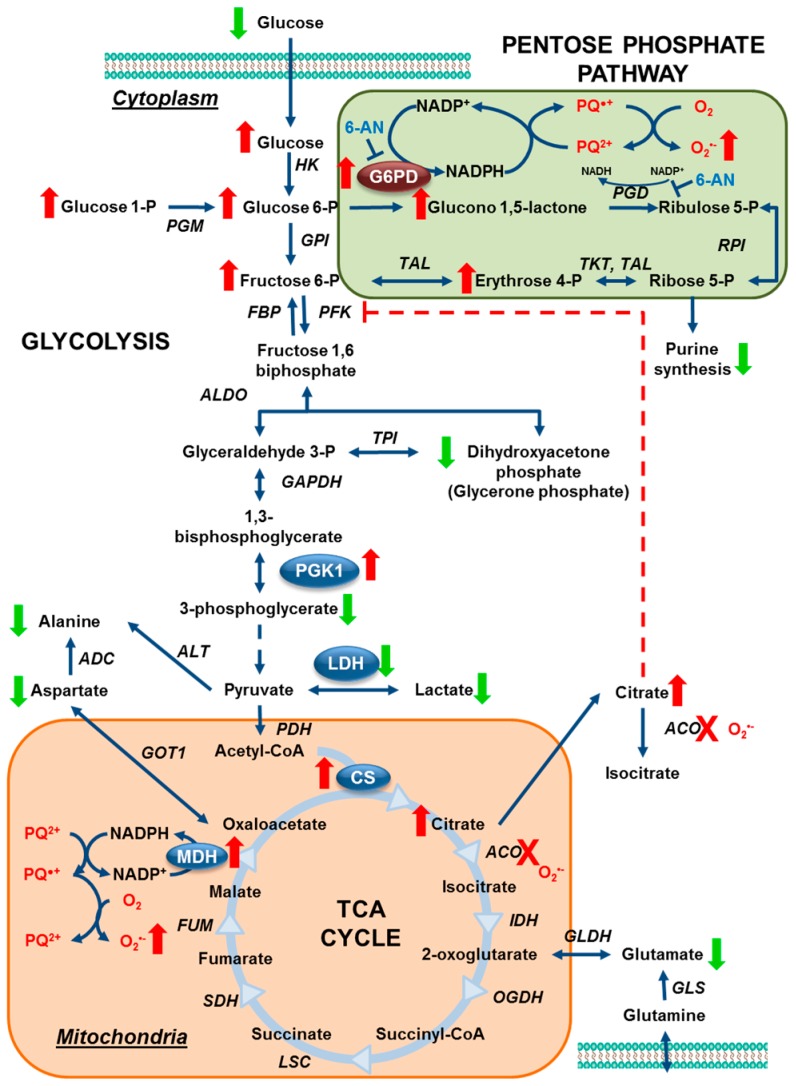
Paraquat hijacks the pentose phosphate pathway to induce oxidative stress and cell death. Our results demonstrate that paraquat induces an increase in the PPP (highlighted in green), which is reflected by an increase in glucose uptake, and in glucose 6-phosphate, glucono 1,5-lactone, erythrose 4-phosphate, and fructose 6-phosphate content (red arrows). In addition, paraquat decreases glycolysis as demonstrated by a decrease in 3-phosphoglycerate, alanine and lactate levels (green arrows). These metabolic changes were also paralleled by: (1) an increase in G6PD (the rate-limiting enzyme in the PPP), and the expression levels of citrate synthase, pyruvate kinases M1/M2; and (2) a decrease in lactate dehydrogenase A/B chains, which participate in glycolysis and the TCA cycle (highlighted in orange). Paraquat also induced an increase in citrate accumulation which is associated to the well-known inhibitory effect on aconitase (highlighted in orange). An abnormal increase in citrate levels has been reported to exert an inhibitory effect on glycolysis by allosteric inhibition of PFK (broken red line), which explains why an increase glucose uptake and impaired TCA cycle is not translated to an upregulation in glycolysis. Modulation of G6PD levels and activity was directly linked to paraquat toxicity and oxidative stress. 6-AN, 6-aminonicotinamide, ACO, aconitase or aconitate hydratase (EC:4.2.1.3; ADC, aspartate 4-decarboxylase (EC:4.1.1.12); ALDO, fructose-bisphosphate aldolase (EC:4.1.2.13); ALT, alanine transaminase (EC:2.6.1.2); CS, citrate synthase (EC:2.3.3.1); FBP, fructose-1,6-bisphosphatase I (EC:3.1.3.11); FUM, fumarate hydratase (EC:4.2.1.2); G6PD, glucose-6-phosphate 1-dehydrogenase (EC:1.1.1.49); GAPDH, glyceraldehyde 3-phosphate dehydrogenase (EC:1.2.1.12); GLDH, glutamate dehydrogenase, (EC: 1.4.1.2); GLS, glutaminase (EC: 3.5.1.2); GOT1, aspartate aminotransferase, cytoplasmic (EC:2.6.1.1); GPI, glucose-6-phosphate isomerase (EC:5.3.1.9); HK, hexokinase (EC:2.7.1.1); IDH, isocitrate dehydrogenase (EC:1.1.1.42); LDH, L-lactate dehydrogenase (EC:1.1.1.27); MDH, malate dehydrogenase (EC:1.1.1.37); OGDH, 2-oxoglutarate dehydrogenase, (EC:1.2.4.2); LSC, succinyl-CoA synthetase (EC:6.2.1.4 6.2.1.5); PC, pyruvate carboxylase (EC:6.4.1.1); PGD, 6-phosphogluconate dehydrogenase (EC:1.1.1.44); PDH, pyruvate dehydrogenase (EC:1.2.4.1); PGK1, phosphoglycerate kinase (EC:2.7.2.3); PGM, phosphoglucomutase (EC:5.4.2.2); PFK, 6-phosphofructokinase 1 (EC:2.7.1.11); RPI, ribose 5-phosphate isomerase A (EC:5.3.1.6); SDH, succinate dehydrogenase (EC:1.3.5.1); TPI, triosephosphate isomerase (EC:5.3.1.1); TAL, transaldolase (EC:2.2.1.2); TKT, transketolase (EC:2.2.1.1). Reproduced with permission from [83].

Human dopaminergic neuroblastoma cells were transduced with adenovirus encoding for human G6PD (AdG6PD) or empty adenovirus (AdEmpty) to further investigate the role of G6PD in paraquat toxicity. G6PD overexpression increased cell death and oxidative death as a result of paraquat treatment, but, importantly, no change was observed when cells were exposed to other toxins (Figure 8a). Conversely, paraquat-induced toxicity, mitochondrial ROS formation, and GSH depletion were reversed when G6PD was inhibited with 6-aminonicotinamide (6-AN) (Figure 8b). Again, 6-AN had no effect on cell death or GSH depletion when neuronal cells were treated with rotenone, MPP^+^, or 6-OHDA instead of paraquat. These observations are consistent with the recycling of NADPH from NADP^+^ by G6PD being uniquely required for the redox cycling of paraquat to produce ROS. In effect, paraquat would be expected to outcompete NADPH-dependent antioxidant systems. As such, unpublished results from our group have demonstrated that depletion of GSH is not prevented by overexpression of GSH reductase. NADPH can also be produced by 6-phosphogluconate dehydrogenase (PGD), malic enzyme (malate dehydrogenase, MDH), and isocitrate dehydrogenase (IDH) [97]. Interestingly, paraquat treatment also increased the expression levels of malate dehydrogenase, which may also contribute to paraquat’s redox cycle in the mitochondria. The observed changes in the metabolome and proteome for human dopaminergic neuroblastoma cells following exposure to paraquat are summarized in Figure 9. Our results suggest that paraquat hijacks the PPP to increase NADPH-reducing equivalents and stimulate paraquat redox cycling, oxidative stress, and cell death. 

### 3.4. Glucose Metabolism Regulates Paraquat Toxicity

**Figure 10:**
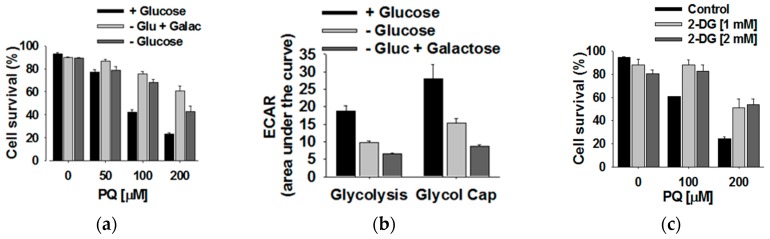
Inhibition of glucose metabolism protects against paraquat toxicity. Rat dopaminergic N27 cells were grown in culture media with or without glucose, or in a glucose-free medium supplemented with galactose. When indicated, cells were treated with PQ or MPP^+^ (2.5 mM) for 48 h in the presence or absence of 2-DG. (**a**,**c**) Cell survival was determined by the simultaneous analysis of plasma membrane integrity (PI uptake) and intracellular GSH content (mBCl fluorescence). Bar graphs represent % s of viable cells (cell survival) and data are means ± SE of at least *n* = 3 independent experiments. (**b**) Glycolysis rates and glycolytic reserve capacity of cells were evaluated by changes in the ECAR sensitive to 2-DG. Glycolysis is observed as an increase in ECAR when switching cells from a glucose-free environment (NG) to a medium containing 10 mM glucose. Glycolytic reserve capacity is determined by addition of oligomycin. Data are means ± SE of at least *n* = 3 independent experiments and are represented with respect to control (+glucose). Two-way ANOVA Holm–Sidak post hoc test: *a*, *p* < 0.05 vs. no PQ or MPP^+^ within the corresponding category of ±glucose, galactose or 2-DG; *b*, *p* < 0.05, vs. +glucose, within the corresponding toxicant treatment. *t*-test: * *p* < 0.05, vs. +glucose. Reproduced with permission from [98].

We further investigated the role of glucose metabolism on paraquat toxicity based on the prior observations that metabolites derived from ^13^C-glucose are uniquely perturbed by paraquat exposure and the hijacking of the PPP. Consequently, glucose availability significantly impacted the survivability of rat dopaminergic mesencephalic cell line N27 following exposure to paraquat. Specifically, glucose deprivation increased cell survival (Figure 10a). Also, replacing glucose with galactose protected cells from paraquat toxicity. Galactose directs metabolism from glycolysis into glutaminolysis and OXPHOS phosphorylation for ATP production. An inhibition in glycolysis for cells grown in glucose-free or galactose-supplemented media was verified by changes in the extracellular medium acidification (ECAR) (Figure 10b). Furthermore, treatment of cells with 2-deoxy-D-glucose (2-DG), which is a hexokinase inhibitor that prevents the production of glucose-6-phosphate, also provided protection from paraquat toxicity (Figure 10c). These results, in total, demonstrate that glucose metabolism contributes to the death of cells following treatment with paraquat at ≥100 μM.

**Figure 11:**
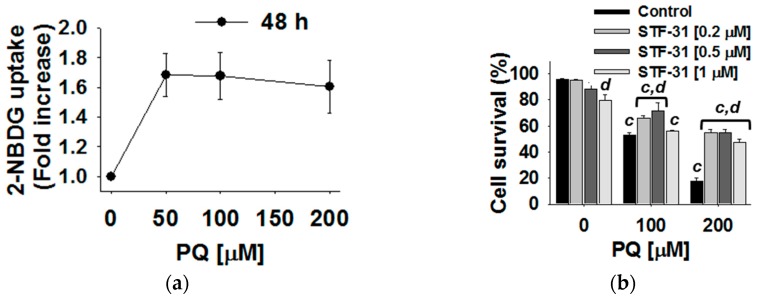
Paraquat increases glucose transport and the translocation of glucose transporters. Cells were treated with PQ for 48 h. (**a**) Glucose transport was evaluated by the uptake 2-NBDG; (**b**) the survival of cells treated with PQ in the presence or absence of STF-31 was determined as explained in Figure 10. The bar graph represents %s of viable cells (cell survival). Data in all graphs are means ± SE of at least *n* = 3 independent experiments. Two-way ANOVA Holm–Sidak post hoc test: c, *p* < 0.05 vs. no PQ within the corresponding ±STF-31 or phlorizin category; d, *p* < 0.05 vs. control (no glucose transport inhibitor) within the corresponding PQ concentration. Reproduced with permission from [98].

The metabolomics analysis indicated both an increase in intracellular glucose and a corresponding decrease in extracellular glucose after exposure to paraquat. These observations suggested that paraquat treatment increases glucose uptake. A fluorescently labeled analog of glucose (2-[N-(7-nitrobenz-2-oxa-1,3-diazol-4-yl) amino]-2-deoxy-D-glucose, 2-NBDG) was used to monitor changes in glucose uptake due to paraquat exposure. Correspondingly, neuronal cells treated with paraquat exhibited a >60% increase in glucose uptake in response to paraquat treatment (Figure 11a), which is consistent with the metabolomics data. Glucose uptake is regulated by a saturable transport system involving the Na+-independent glucose transporters (GLUT), and the Na+-dependent glucose transporters (SGLT). Exposure to paraquat resulted in a significant increase in the translocation of SGLT1 and GLUT4 transporters to the plasma membrane. Consequently, inhibiting glucose uptake with STF-31, a GLUT inhibitor, (Figure 11b) or ascorbic acid, a competitive inhibitor of glucose, decreased paraquat toxicity. Again, these results clearly demonstrate that glucose metabolism is an important contributor to paraquat-induced cell death.

### 3.5. Paraquat Induced Metabolic Dysfunction in the Mice Midbrain and Striatum 

**Figure 12:**
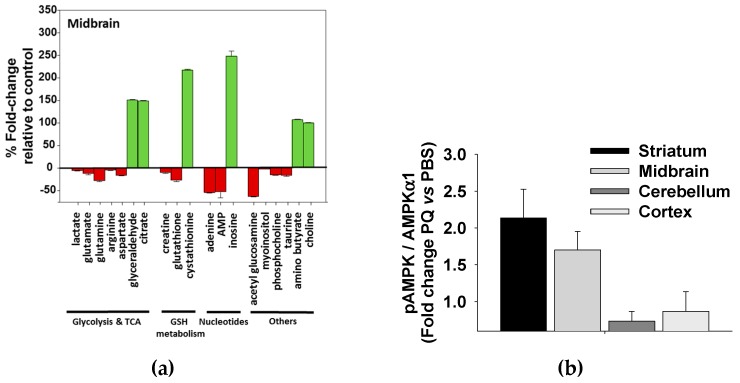
C57Bl/6J mice were exposed chronically to paraquat (PQ). One week after the final injection of PQ or PBS, animals were euthanized to isolate metabolites from the midbrain, striatum and cortex regions. Integrated positive and negative-ion DI-ESI-MS and 1D ^1^H NMR were used to characterize the alterations in the metabolic profiles of midbrain, striatum, and cortex regions from control and PQ-treated mice. (**a**) The percent fold-change for metabolites contributing to class separation as identified from OPLS-DA back-scaled loadings plots are plotted. The percent fold changes are all significant (*p* < 0.05) based on a paired t-test. The green bars indicate metabolites with a fold-increase after PQ treatment, whereas red bars indicate that a metabolite decreased after PQ treatment. Reproduced with permission from [98]. (**b**) Changes in the levels of phosphorylated (p) AMPKα1 induced by PQ were evaluated by western-bot (WB). Bar graphs represent the densitometry analysis of the corresponding WBs from three independent replicas. Data are represented as fold change vs. the indicated control. Reproduced with permission from [98].

To further substantiate the validity of our in vitro findings that paraquat uniquely perturbs the metabolome of dopaminergic neurons, we evaluated the metabolic dysfunction induced by chronically treating C57BI/6 mice with paraquat. We observed that paraquat only induced statistically significant changes to the metabolomes of the midbrain, which is the location of the substantia nigra and the loss of dopaminergic neurons associated with PD; and to the striatum, which receive dopaminergic signals from midbrain and controls motor function. Importantly, we observed changes to metabolites associated with glycolysis (e.g., lactate), the TCA cycle (e.g., glutamate), and GSH metabolism (e.g., glutathione), consistent with our in vivo results (Figure 12a). Paraquat was again observed to induce a large accumulation of citrate consistent with the proposed inactivation of aconitase. AMP-activated protein kinase (AMPK) is a metabolic master regulator that includes regulating glucose uptake and, like other kinases, is regulated by phosphorylation (pAMPK) [99]. Consequently, chronic paraquat treatment also resulted in a significant increase in pAMPK and its substrate acetyl-CoA carboxylase (pACC) only in the midbrain and striatum of C57BI/6 mice (Figure 12b). These results further corroborate the observation that paraquat modulates glucose metabolism and that the midbrain and striatum are selectively sensitive to paraquat toxicity.

## 4. Synergy of α-Synuclein Genetic Mutations and Paraquat Toxicity

### 4.1. α-Synuclein Potentiates Paraquat Toxicity and Metabolic Dysfunction

**Figure 13:**
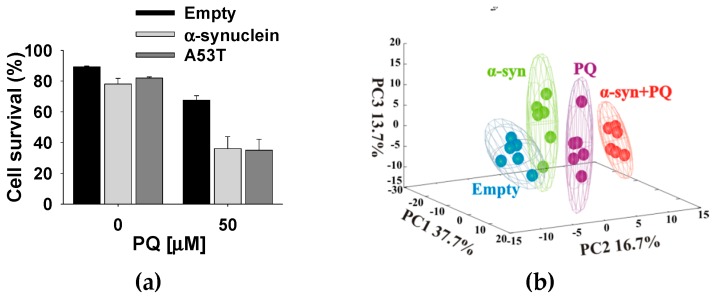
α-Synuclein potentiates the metabolic dysfunction and toxicity induced by paraquat (PQ). N27 dopaminergic cells were transduced for 24 h with empty adenoviruses or adenoviruses encoding either WT or mutant A53T α-synuclein (6 MOI). (**a**) Cell survival after exposure to PQ for 48 h was determined as explained in Figure 10. Bar graph represents percentage of viable cells (cell survival) and data are means ± SE of at least *n* = 3 independent experiments. (**b**) Cells were treated with 25 µM PQ for 24 h. Metabolites were extracted for NMR/MS metabolomics. 3D MB-PCA scores plot shows the changes in the metabolome based on distances between groups. The ellipsoids correspond to the 95% confidence limits from a normal distribution for each cluster. Six independent samples of metabolic extract were used for the MB-PCA multivariate analysis. Reproduced with permission from [98].

**Figure 14:**
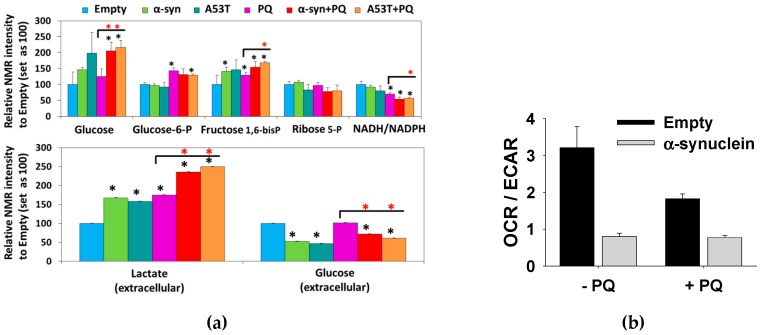
α-Synuclein potentiates paraquat induced metabolic changes. (**a**) Paraquat N27 dopaminergic cells were transduced for 24 h with empty adenoviruses or adenoviruses encoding either WT or mutant A53T α-synuclein (6 MOI). Cells were treated with 25 µM PQ for 24 h. Metabolites were extracted for NMR metabolomics. 2D ^1^H−^13^C HSQC NMR spectra from ^13^C glucose labeling experiments were used to evaluate the intracellular metabolic changes shown by the MB-PCA multivariate analysis. Data represent the mean of 3 independent replicates. * *p* < 0.05 vs. empty. (**b**) Cells were treated with 25 µM PQ for 12 h. Basal OCR and ECAR rates were determined after 1 h of incubation of cells with fresh medium. Data represent means ± SE of at least *n* = 3 independent experiments. Reproduced with permission from [98].

PD is a probable multifactorial disease, in which exposure to an environmental toxin is likely one component in the development and progression of the disease. Overexpression and aggregation of α-synuclein and the subsequent formation of Lewy bodies are hallmarks of PD. Consequently, is there a synergistic relationship between α-synuclein and exposure to paraquat? The overexpression of wild-type (WT) α-synuclein or an A53T mutant (familial PD mutant [9,10]) did not result in any significant change in viability (Figure 13a). Conversely, treatment with paraquat resulted in a significant increase in cell death and a major perturbation in the metabolome (Figure 13). Specifically, the combination of paraquat and α-synuclein overexpression produced a more dramatic change in the metabolome than either paraquat treatment or α-synuclein overexpression alone. Importantly, the same results were obtained if either WT or A53T α-synuclein was overexpressed. A detailed analysis of the metabolic changes was then made using 2D ^1^H-^13^C HSQC experiments and following the ^13^C-carbon distribution resulting from ^13^C-glucose supplemented media (Figure 14a). Overexpression of α-synuclein and paraquat exposure resulted in an enhanced glucose accumulation, an impairment in glycolysis, and a reduction in glycolytic capacity and mitochondrial respiration (OCR/ECAR ratio) (Figure 14b). 

### 4.2. Glucose Metabolism Contributes to Synergistic Toxicity between Paraquat and α-Synuclein

**Figure 15:**
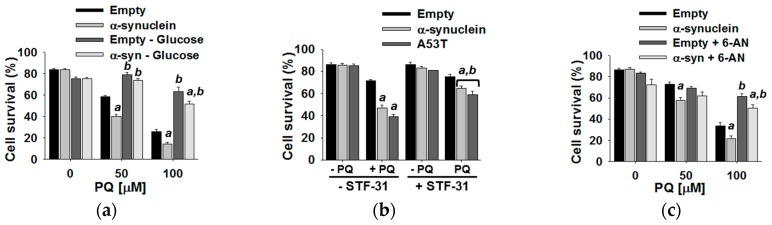
Glucose metabolism and the PPP regulate the toxic synergism of α-synuclein and paraquat (PQ). N27 dopaminergic cells were transduced for 24 h with empty viral particles or adenoviruses encoding either WT or mutant A53T α-synuclein (6 MOI). After transduction, cells were treated with PQ (50 µM in b) for 48 h in the presence or absence of glucose. (**a**) STF-31 (b, 0.5 mM), or 6-AN (c, 1 mM). Cell survival was determined as explained in Figure 10. Bar graphs represent % s of viable cells (cell survival) and data are means ± S.E.M. of at least *n* = 3 independent experiments. Two-way ANOVA Holm–Sidak post hoc test was done for each PQ concentration independently: a, *p* < 0.05, vs. empty, within the corresponding category of ±glucose (**a**) ±STF31; (**b**) ±6-AN (**c**); b, *p* < 0.05, vs. +glucose (**a**), -STF31 (**b**), or -6-AN (**c**), within the corresponding category of Empty or α-synuclein. Reproduced with permission from [98].

As described in Section 3.4, glucose metabolism was shown to regulate paraquat toxicity. Specifically, glucose deprivation protected dopaminergic cells from paraquat-induced cell death. Additionally, replacing glucose with galactose or the inhibition of glycolysis with 2-DG were also observed to prevent cell death from paraquat exposure (Figure 10). Furthermore, paraquat treatment was shown to cause an increase in glucose uptake, and inhibiting glucose uptake was also shown to provide protection against paraquat toxicity (Figure 11). Finally, the inhibition of PPP by 6-AN (Figure 8b) significantly increased cell survival following exposure to paraquat. These results raise the question: does glucose metabolism play a similar role and contribute to the observed synergistic toxicity between paraquat and α-synuclein? To address this question, we repeated all of the above experiments with the addition of the overexpression of WT α-synuclein or an A53T mutant. Glucose deprivation, the inhibition of glucose uptake, and the inhibition of the PPP were all observed to eliminate the stimulatory effect of α-synuclein on paraquat toxicity (Figure 15). These results, in total, provide strong evidence that glucose metabolism and signaling are critically involved in the toxic synergism of paraquat exposure and the overexpression of α-synuclein. Specifically, α-synuclein potentiates paraquat toxicity by impairing energy metabolism, glycolysis, and mitochondrial respiration. 

## 5. Conclusions

PD is currently believed to be a multifactorial disease, in which age, genetics, and environmental toxins are all considered significant risk factors. The overexpression or mutation of α-synuclein [9,10] has been identified as a major genetic factor associated with PD. In fact, the formation of α-synuclein aggregates or Lewy bodies in dopaminergic neurons are the primary clinical sign of the disease. Lewy bodies are correlated with neuronal loss and cognitive impairment, which are key symptoms of PD. The biological function of α-synuclein is currently unknown, and while a variety of factors have been shown to induce fibril formation, oxidative stress [49] is an important contributor. Thus, mitochondrial dysfunction, energy failure, and redox imbalance induced by environmental toxicants may be the primary mechanism leading to α-synuclein misfolding and aggregation in PD.

**Figure 16:**
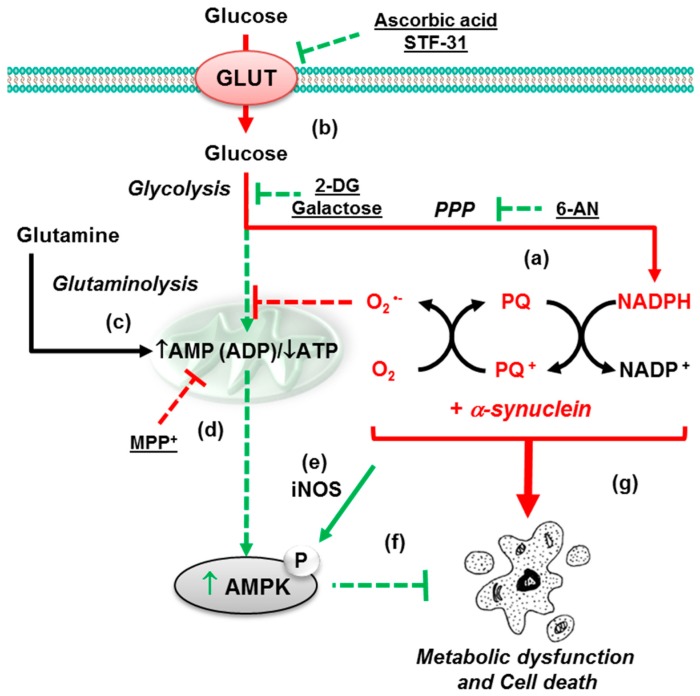
Glucose metabolism and AMPK signaling regulate the toxicity of paraquat + α-synuclein. We have demonstrated that PQ hijacks the PPP to use NADPH electrons for redox cycling and to induce cell death. (**a**) We also revealed that glucose metabolism/transport contributes to PQ-induced dopaminergic cell death, as evidenced by the protective effects of STF-31, AA (GLUT-like transport inhibitors) and 2-DG (glucose metabolism inhibitor). (**b**) Furthermore, we present evidence that the stimulation of glutamine metabolism via the TCA cycle by galactose supplementation also protects against PQ; (**c**) in contrast, glucose metabolism protected against the mitochondrial complex I inhibitor MPP^+^ while sole reliance on glutamine metabolism induced by galactose supplementation sensitized cells to MPP^+^-induced cell death; (**d**) PQ-induced AMPK signaling is dependent on iNOS; (**e**) AMPK signaling activated in response to PQ or glucose deprivation exerted a protective effect against PQ; (**f**) overexpression of α-synuclein stimulated PQ toxicity (gene-environment interaction), metabolic dysfunction and AMPK activation; (**g**) reproduced with permission from [98].

Exposure to environmental toxins, such as herbicides or pesticides, has been correlated with an increase in the incidence of PD and has been shown to induce PD-like symptoms. Specifically, environmental toxins have been shown to induce cell death of dopaminergic neurons primary through an increase in ROS. Consequently, the loss of neurons in the substantia nigra of the midbrain is a hallmark of PD. We have demonstrated that paraquat, rotenone, 6-OHDA, and MPP^+^ have distinct molecular mechanisms leading to dopaminergic neuron cell death. We have shown that paraquat hijacks the NADPH from the PPP to redox cycle, induces oxidative damage, and impairs antioxidant defenses. Furthermore, paraquat increases glucose transport and carbon flux to the PPP (Figure 16). Finally, paraquat impairs the TCA cycle, which leads to the accumulation of citrate and impairment of glycolysis. Critically, we have demonstrated a clear toxic synergy between paraquat and α-synuclein. α-Synuclein impairs glycolysis and upregulates glucose transport. This channels carbon flux to the PPP to increase paraquat’s redox cycling and ROS formation. In effect, α-synuclein feeds the production of ROS that leads to its own misfolding and aggregation. Consequently, glucose metabolism plays an important role in the molecular mechanism of PD since it regulates the effect of α-synuclein on paraquat toxicity. The inhibition of glucose transporters prevents the potentiation of paraquat toxicity by α-synuclein. Also, the inhibition of the PPP protects against this synergistic toxicity. Consistent with the cell-based assays, we also observed that paraquat selectively induced metabolic dysfunction in the midbrain and striatum of C57Bl/6 mice. Overall, glucose metabolism and AMPK contribute to dopaminergic cell death, induced by PQ and α-synuclein interactions. Central carbon metabolism and metabolic dysfunction/signaling are important contributors to dopaminergic cell death induced by gene–environment interactions. 

## 6. Future Perspectives

The work presented herein provides some insights into potential underlying molecular mechanisms that lead to PD, specifically with regards to the herbicide paraquat. It also provides valuable evidence in support of a gene–environment interaction that may increase the likelihood of developing PD. Nevertheless, the molecular mechanism attributed to other environmental toxins is still currently unknown. Additionally, the biological role in PD for other genetic alterations (i.e., Parkin, DJ-1, PINK1, and LRRK2) requires further investigation. Even in the case of α-synuclein, the mechanism by which α-synuclein interacts with paraquat to induce cell death is still unclear. For example, it is still uncertain if α-synuclein aggregates are a direct cause of neuronal cell death or if the formation of Lewy bodies is a protective mechanism. Similarly, while some evidence was provided that cell-based results are consistent with in vivo studies, substantial effort is still required to verify if any of these processes are related to the development of PD in human patients. Consequently, there is more that we still do not know about the molecular processes that lead to the development of PD. However, this study clearly demonstrated the inherent value of combining metabolic studies with traditional cell biology to explore the molecular mechanisms associated with Parkinson’s disease. Metabolomics is expected to continue to play an important role in further PD studies.

While avoiding environmental toxins is obvious advice, it is particularly pertinent for individuals with a genetic predisposition to developing PD. However, we are still far from identifying the environmental agents that are a current concern to the public and are also linked to an increased risk in developing PD. For example, most of the research to date has been focused on pesticides (paraquat and rotenone) with a strong epidemiological association with PD, but whose usage is restricted and thus are not currently a major risk for human populations. More importantly, it is clear that a single environmental exposure will not (at least by itself) cause PD, which complicates the further identification of gene–environment interactions, which, together with aging, may trigger progressive neurodegeneration. 

Since our research strongly suggests that glucose metabolism contributes to PQ toxicity and to the synergistic effect between PQ and α-synuclein overexpression, a low-carbohydrate diet may be beneficial to preventing the development and progression of PD [100]. In fact, a ketogenic diet (low carbohydrate/high fat) has been reported to exert protective effects in PD [101]. To date, there are no successful therapies to treat or prevent PD. As a result, α-synuclein has garnered a tremendous amount of attention as a therapeutic target with a number of potential treatments in development or in clinical trials [102]. These potential drugs are designed to increase α-synuclein clearance, prevent its aggregation, or inhibit post-translational modifications, but no clinical successes have been observed as of yet. Correspondingly, an alternative strategy may be to directly target the metabolic processes “hijacked” by toxins associated with PD. FDA-approved metabolic inhibitors have been safely used as drugs in humans for decades [103]. While broad-based antioxidant therapies have not had much success in treating PD [104], a focused effort on developing drugs that inhibit a specific target of a toxin, such as G6PD in the case of PQ, may be a viable alternative. Furthermore, a personalized therapy based on a patient’s history of toxin exposure coupled with a ketogenic diet may prove to be a successful approach to prevent the progression of the disease. 

## Figures and Tables

**Table 1:** Prevalence of PD in Nebraska [35].

	Age (Years)		
**Nebraska PD Prevalence**	**60–70**	**70–80**	**80+**
(Rates per 100,000)			
Men	406	1794	4248
Women	298	991	2069

## Abbreviations

The following abbreviations are used in this manuscript:

1D	one-dimensional
2D	two-dimensional
3D	three-dimensional
2-DG	2-deoxy-D-glucose
2-NBDG	2-[N-(7-nitrobenz-2-oxa-1,3-diazol-4-yl)amino]-2-deoxy-D-glucose
6-AN	6-aminonicotinamide
6-OHDA	6-hydroxydopamine
α-syn	α-synuclein
AA	ascorbic acid
ACO	aconitase
AD	Alzheimer’s disease
ADC	aspartate 4-decarboxylase
AdEmpty	empty adenovirus
AdG6PD	adenovirus encoding for human G6PD
ADP	adenosine diphosphate
ALDO	fructose-bisphosphate aldolase
ALT	Alanine transaminase
AMP	adenosine monophosphate
AMPK	AMP-activated protein kinase
AMPKα1	AMP-activated protein kinase α1
ANOVA	analysis of variance
ATP	adenosine triphosphate
BBB	blood brain barrier
CS	ATP-citrate synthase
CV-ANOVA	ANOVA of the cross-validated residuals
DHAP	dihydroxyacetone phosphate
DI-ESI-MS	direct infusion-electrospray ionization mass spectrometry
DNA	deoxyribonucleic acid
ECAR	extracellular acidification rate
ECV	extraction voltage
FBP	fructose-1,6-bisphosphatase I
FUM	fumarate hydratase
G6PD	glucose-6-phosphate dehydrogenase
GAPDH	Glyceraldehyde 3-phosphate dehydrogenase
GLDH	Glutamate dehydrogenase
GLS	Glutaminase
GLUT	Na+-independent glucose transporters
GOT1	Aspartate aminotransferase, cytoplasmic
GPI	Glucose-6-phosphate isomerase
GSH	reduced glutathione
GSSH	oxidized glutathione
HK	hexokinase
HSQC	heteronuclear single quantum coherence
IDH	isocitrate dehydrogenase
iNOS	inducible nitric oxide
LDH	L-lactate dehydrogenase
LRRK2	leucine-rich repeat kinase 2
LSC	succinyl-CoA synthetase
mBCl	monochlorobimane
MB-PCA	multiblock principal component analysis
MB-PLS	multiblock partial least squares
MDH	malate dehydrogenase
Mn	manganese
MOI	multiplicity of infection
MPP^+^	1-methyl-4-phenylpyridinium
MPTP	1-methyl-4-phenyl-1,2, 3, 6-tetrahydropyridine
MS	mass spectrometry
NAC	non-amyloid-β component of AD amyloid plaques
NADP	nicotinamide adenine dinucleotide phosphate
NADPH	nicotinamide adenine dinucleotide phosphate hydrogen
NG	glucose free environment
NMR	nuclear magnetic resonance spectroscopy
OCR	oxygen consumption rate
OGDH	2-oxoglutarate dehydrogenase
OPLS	orthogonal projection to latent structures
OXPHOS	oxidative phosphorylation
pACC	phosphorylated acetyl-CoA carboxylase
pAMPK	phosphorylated AMP-activated protein kinase
PBS	phosphate buffered saline
PC	pyruvate carboxylase
PCA	principal component analysis
PD	Parkinson’s disease
PDH	pyruvate dehydrogenase
PET	positron emission tomography
PFK	6-phosphofructokinase 1
PGD	6-phosphogluconate dehydrogenase
PGK1	phosphoglycerate kinase 1
PGM	phosphoglucomutase
PI	propidium iodide
PINK1	phosphatase and tensin homolog (PTEN)-induced kinase 1
PLS	partial least squares
PPP	pentose phosphate pathway
PQ	paraquat
PSC	phase-scatter correction
RNA	ribonucleic acid
ROS	reactive oxygen species
RPI	ribose 5-phosphate isomerase A
SCV	sampling cone voltage
SDH	succinate dehydrogenase
SE	standard error
SGLT	Na^+^-dependent glucose transporters
SNARE	soluble N-ethylmaleimide-sensitive factor (NSF) attachment protein receptor
STF-31	4-[[[[4-(1,1-dimethylethyl)phenyl]sulfonyl]amino]methyl]-N-3-pyridinyl-benzamide
SUS	shared and unique structures
TAL	transaldolase
TCA	tricarboxylic acid
TKT	transketolase
TPI	triosephosphate isomerase
US	United States
UDP	uridine diphosphate
UMP	uridine monophosphate
WB	Western blot
WT	wild-type
